# A systematic approach to the planning, implementation, monitoring, and evaluation of integrated health services

**DOI:** 10.1186/1472-6963-13-168

**Published:** 2013-05-06

**Authors:** Heidi W Reynolds, Elizabeth G Sutherland

**Affiliations:** 1 MEASURE Evaluation, Carolina Population Center, University of North Carolina at Chapel Hill, 206 W. Franklin Street, 2nd Floor, CB 8120, Chapel Hill, NC 27516, USA

**Keywords:** Integration, Health services, Monitoring and evaluation, Developing countries, Conceptual framework

## Abstract

**Background:**

Because of the current emphasis and enthusiasm focused on integration of health systems, there is a risk of piling resources into integrated strategies without the necessary systems in place to monitor their progress adequately or to measure impact, and to learn from these efforts. The rush to intervene without adequate monitoring and evaluation will continue to result in a weak evidence base for decision making and resource allocation. Program planning and implementation are inextricability linked to monitoring and evaluation. Country level guidance is needed to identify country-specific integrated strategies, thereby increasing country ownership.

**Discussion:**

This paper focuses on integrated health services but takes into account how health services are influenced by the health system, managed by programs, and made up of interventions. We apply the principles in existing comprehensive monitoring and evaluation (M&E) frameworks in order to outline a systematic approach to the M&E of integration for the country level. The approach is grounded by first defining the country-specific health challenges that integration is intended to affect. Priority points of contact for care can directly influence health, and essential packages of integration for all major client presentations need to be defined. Logic models are necessary to outline the plausible causal pathways and define the inputs, roles and responsibilities, indicators, and data sources across the health system. Finally, we recommend improvements to the health information system and in data use to ensure that data are available to inform decisions, because changes in the M&E function to make it more integrated will also facilitate integration in the service delivery, planning, and governance components.

**Summary:**

This approach described in the paper is the ideal, but its application at the country level can help reveal gaps and guide decisions related to what health services to prioritize for integration, help plan for how to strengthen systems to support health services, and ultimately establish an evidence base to inform investments in health care. More experience is needed to understand if the approach is feasible; similarly, more emphasis is needed on documenting the process of designing and implemented integrated interventions at the national level.

## Background

Integration, and integrated health services specifically, are widely being promoted as a way to gain efficiencies, meet clients’ varied health needs and ultimately improve health outcomes. The U.S. Global Health Initiative calls for both “upstream” and “downstream” integration to coordinate and integrate health interventions [1]. The Global Health Initiative, Paris Declaration, World Bank Sector Wide Approaches, the International Health Partnership Plus ^a^ are models of “upstream” integration and coordination at the national level and higher, where donors, partner governments and other implementers harmonize financing and work together to develop and implement national health plans [1-4]. In the U.S. President’s Emergency Plan for AIDS Relief (PEPFAR) five-year strategy, there is an important priority for “downstream” integration of family planning and HIV services at the health service and individual levels “so that women living with HIV can access necessary care, and so that all women know how to protect themselves from HIV infection” [5].

Although these are some of the more recent endorsements of integration, in 1978, the Declaration of Alma Ata promoted a comprehensive approach to health starting with primary health care [6]. While integration is promoted for its potential to provide greater access to more comprehensive care and to create health system efficiencies, there is a lack of consensus about the concept of integration and how to operationalize integration, and the field is lacking empirical evidence for effective models to guide decision making. More information is needed from integrated program designs about specifically what are the changes being made and to what program element (such as governance, funding, service delivery organization, etc.) [7].

Strong monitoring and evaluation (M&E) systems can provide the information needed to assess progress, generate information for program management and decision making, and produce evidence of impact on health outcomes to inform replication and scale up [8]. In the last decade, monitoring systems and indicators have proliferated in health, particularly for HIV, while research and evaluation has been neglected or implemented *post-hoc*[9]. Moreover, how building M&E systems from the outset alongside program planning can benefit program planning is not well understood or appreciated. However, new funding for HIV and AIDS control through global health initiatives has created opportunities for increased multi-sectoral participation, political commitment, and transparency in M&E systems [10], and there is growing momentum to ensure that process and outcome or impact evaluations are planned from the outset to inform decision making [11,12].

To respond to the need for evidence of effective integrated services to inform the design and operationalization of integration, in this paper, we prioritize and organize existing M&E principles in to a systematic approach specifically relevant for health service integration initiatives. The primary audience for this approach is those program planners working at the national level. The approach is intended to help decision making related to what health services to prioritize for integration, what systems to strengthen to support integrated health services, and what data to collect to best monitor and evaluate integrated health services. Ultimately, the approach is intended to help establish an evidence base of what works to help inform decisions about these investments at both the national and international levels.

The approach outlined in this paper represents the ideal. In reality, each country will have performed each step at differing levels of depth and breadth. Thus, applications of the approach will reveal gaps. The approach is also iterative, in revisions can take place in earlier steps based on information gained in later steps.

## Discussion

### Definitions of health service integration

Although integration is a broad term, many definitions highlight service delivery combinations. The World Health Organization (WHO) defines integration as “combining different kinds of … services or operational programs to ensure and maximize collective outcomes. It would include referrals from one service to another and is based on the need to offer comprehensive services” [13]. Although the WHO definition is specific for reproductive health and HIV integration, it is applicable to other combinations.

The ultimate aim is to improve health outcomes, and health services are the most proximate function to that end. *Health services* include the infrastructure, human resources, and supplies and technologies necessary to provide care to clients [14]; thus, all those activities are affected by integrated health services. Integrated health services can be characterized as vertical, i.e., between different levels of service delivery from the community-level to clinic and hospital-level, or horizontal, i.e., with providers or organizations working at the same levels of service delivery. The mechanisms of integrated care delivery include referrals, coordinating care from multiple providers during single visits, and multiple services available from a single provider during a single visit [15]. The pathway through which integrated health services are expected to improve client outcomes is by improving the continuum of care for clients (see WHO, 2008 for a definition of continuum of care) [16].

Health services operate within the environment of the larger health system. The *health system* is a complex system comprising a set of functions that generally include leadership and governance, financing, planning, commodities, workforce, service delivery and information systems with the ultimate goal to improve health outcomes [14,17]. It is through improvements in the health system functions where the intermediate goals of reduced fragmentation and duplication and increased efficiency and acceptability are reached. Health system functions play an important role in the success or failure of integrated health services. Absent, weak, or poorly defined policies and guidelines; human resource constraints including inadequate number and distribution of well trained staff; irregular and inadequate supplies of drugs and materials because of weak supply chain systems; delayed release of funds and failure to allocate resources to ensure service delivery functionality; poor planning, management, and supervision; lack of linkages between facilities and community; and weak, incomplete or siloed health information systems have all been shown to limit the potential effectiveness of integrated health interventions [18,19].

Atun and colleagues have addressed integration of interventions into the health system, and in their framework they define how interventions are more or less integrated in to health system functions (20). An intervention is considered fully integrated into the health service function if the intervention was available from the same multi-purpose provider. Partial integration is characterized by shared responsibilities across providers or through service linkages, and no integration is indicated by single purpose health workers with no linkages to other services.

According to Atun and colleagues (20), the M&E function of a health intervention is considered fully integrated if the responsibility for M&E rests with institutions, such as Ministries of Health, that have overall responsibility for M&E in the health system. An integrated M&E function for a new health intervention includes use of shared indicators, integrated data collection, recording, analysis, and reporting systems [20]. Partial integration is characterized by shared responsibility between the government institutions and international partners, whereas no integration of M&E systems are those instances when M&E is carried out by independent institutions or donors.

Health system functions do not necessarily need to be integrated in and of themselves in order to facilitate integrated services. For example, it is not a pre-requisite for drug and commodity procurement systems and supply chain management to be integrated; however, for a client who needs treatment for both HIV and TB, the system should appear to be seamless to the client. Similarly, the M&E function does not necessarily need to be fully integrated as long as the information needed to inform program planning is available. However, changes in the M&E function to make it more integrated will facilitate integration in the service delivery, planning and governance functions. For example, to be able to ensure a client’s continuum of care at a point in time or across the life course must include functional systems that link clients’ health information, so that clients do not bear the (possibly unrealistic) expectation of being the primary source of complete and accurate health history for each new provider at each new point of contact. A single, provider-accessible record may be one way to capture clients’ health information over time. Further, a more integrated M&E function will facilitate integrated planning and governance functions because the M&E data will be available to local and national decision makers to inform program planning, management and supervision [20]

### Monitoring and evaluating in the context of integrated health services

The traditional M&E process is a series of important decision points: Identify the problem, plan the response to the problem, monitor implementation of the response, collect and analyze data to revise the response as needed and assess the effectiveness of the response [8]. These steps are evident in existing frameworks such as the UNAIDS’ Public Health Questions Approach to HIV Monitoring and Evaluation and Bryce and colleagues’ approach to evaluating the scale up for maternal and child survival (22–24). The M&E process is iterative, where information gained in the latter steps can be used to go back and improve program responses in earlier steps. The frameworks are also useful to help identify information needs and gaps and to plan to fill those gaps. Another central feature are logic models or program impact pathways that describe the theoretical pathways of influence of the intervention, and are useful to identify and define key indicators and data sources to measure the effects [21]. Programs are planned using data, and the data collected are informed by program plans.

**Figure 1:**
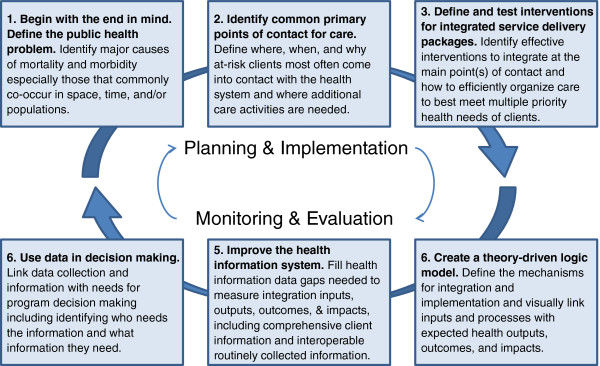
The 6-step systematic approach to the M&E of integration.

The following 6-step systematic approach to the M&E of integration presents what happens in the ideal (Figure 1). It is informed by the basic M&E questions for national level M&E systems and M&E best practices embodied in existing frameworks [8,22-24], but the approach goes beyond those basic M&E questions in order to make this process appropriate for integrated services. The steps in the approach are:

1. Begin with the end in mind. Define the public health problem.

2. Identify common primary points of contact for care.

3. Define and test interventions for integrated service delivery packages.

4. Create a theory-driven logic model.

5. Improve the health information system.

6. Use data in decision making.

#### Begin with the end in mind. Define the public health problem

Understanding the main causes of death and disease in a country’s population and how those vary by sub-populations or geography is a fundamental first step to defining any programmatic response. The scope and intensity of the health problems and people affected are determined through monitoring deaths and births, surveillance, and household surveys [25]. An integration scenario addresses the same national health priorities, targets, and goals identified though mortality monitoring, surveillance, and surveys. Determining the programmatic response, however, requires a systematic examination of the full range of health concerns and recognition of which services could work together to mutually address the specific public health priorities, targets, and goals. For example in three countries (Zambia, Kenya, and Ethiopia) that are experiencing relatively high levels of HIV burden in general, there has also been recognition of high levels of HIV and TB co-infection rates (70%, 48%, and 19%, respectively) [26]. These three countries, recognizing that a reduction of HIV and TB incidence are national health priorities and that these health challenges are largely affecting a common population, have prioritized HIV and TB integration and produced national HIV/TB integration policies and guidelines and convened national coordinating committees [27-32].

#### Identify common primary points of contact for care

Common primary points of contact include:

•antenatal or maternity care

•HIV counseling and testing

•curative or ambulatory services

•child wellness and

•community based models of care, such as home visits or peer-to-peer contact

Contact with health care ranges from community-based care to primary and tertiary health facilities. Community-based care is particularly appropriate when access to facility-base care is limited by geography, stigma, or other factors.

When clients access care, they are met by a specific activity or set of activities (i.e., *health interventions*) intended to improve some aspect of health [33]. For example, a pregnant woman seeking routine essential antenatal care will likely receive: confirmation of pregnancy, detection of problems such as anemia or hypertension, tetanus immunization, counseling for care at home such as nutrition, birth planning, and syphilis testing [34]. Women and babies with diseases or complications may receive additional or other specialized care. Provision of some interventions may be limited in availability to certain levels of care and types of providers. For example, insecticide treated nets may be widely available, but treatment for syphilis requires a provider with special skills at a higher level facility. This kind of uneven service availability, without the benefit of integration, negatively affects the continuum of care available to clients.

Based on the findings from step 1, where the scope and intensity of the full range of health problems and people affected have been assessed and prioritized, program planners need to identify point(s) of contact where additional care activities are needed to ensure that clients’ health needs are comprehensively addressed. Taking again the example of the countries of Zambia, Kenya and Ethiopia, because of the high burden of HIV and co-infection, all three have prioritized HIV testing for TB clients and screening and referral to TB for people in HIV care and treatment [27-29,32]. Regarding the antenatal care example, in settings with high HIV prevalence, antenatal care may reach large numbers of women of reproductive age at risk of HIV or living with HIV and at risk of passing the virus to their infants. For all women entering antenatal care, WHO has added HIV counseling and testing to the set of essential services [35]. These examples all illustrate how interventions based on *national health priorities* and arranged around *specific points of contact* can result in an integrated approach to health services.

In settings where there is little integration in general or at a specific point of contact, initial efforts to add interventions may occur at the program or pilot level until enough evidence is generated to inform national implementation. For example, women living with HIV are at increased risk for cervical dysplasia; but in developing country settings, few women have access to cancer screening. Mwanahamuntu and colleagues argue that the HIV care and support platform is an ideal setting to screen women who are also at risk of cervical cancer [36]. If work to identify cost-effective strategies for screening and cancer prevention is successful, then countries prioritizing cancer prevention will use this evidence to inform their national policies of a specific set of activities and points of contact for services.

It is unrealistic to expect that all entry points will offer comprehensive or even complete care. Priorities for *where* (what service delivery point) to integrate will have to be determined based on a country assessments of the national health priorities, epidemiology and health service use defined above. Other decision points include *what* additional interventions and services to offer and the mechanism of access (e.g., referral or onsite provision). As suggested by the cervical cancer example, *what* will also be informed by the operational evidence of the screening, prevention, or treatment options available to integration and whether these technologies are feasible and cost-effective. Another decision point is *how* organizations, providers, and others will work together to coordinate care, including informal interactions and information sharing, collaboration through mutual agreements or guidelines, more formal coordination mechanisms such as common management and supervision, oversight committees, merged services, etc. [14]. Other health system considerations need to be factored into to supporting integrated service delivery such as whether training or decision tools are needed to strengthen human resources. Research and evaluation can help understand the optimal intervention packages and mechanisms of delivery. Case studies can be useful to document how organizations and others coordinate care and what changes to health system functions are necessary.

#### Define and test interventions for integrated service delivery packages

As additional interventions necessary to meet clients’ comprehensive care needs are identified and integrated by care entry point, and evidence is generated about the most effective set of integrated interventions, as well as on the most successful techniques for implementing these interventions, then standardized interventions packages and guidance can be developed at the national or international levels. The defined intervention package will also help identify and standardize critical indicators that should be measured for monitoring.

At the international level, there are already a number of documents providing guidance on integrated service delivery activities. As mentioned earlier, WHO has produced guidance reflecting integrated care for pregnant women and their infants, during the pregnancy and postpartum intervals, and throughout the various levels of care [34,35]. For key populations (also known as most-at-risk populations or MARPs), USAID recommends a comprehensive service package that aims to address behavioral, biomedical, and structural HIV prevention interventions. It is not only integrated from the sense that it contains services from a range of health sectors (e.g. screening and care for HIV, STIs, family planning, and primary care), but it is also a good example of integrating clinical and non-clinical and community-based approaches to improving a particular priority health outcome [37]. PEPFAR has similarly produced guidance on a recommended package of integrated prevention of mother-to-child of HIV/pediatric HIV/maternal and neonatal and child health services and health system strengthening activities [38].

International guidance documents come from a combination of empirically supported as well as theory-driven approaches. They are developed though multilateral coordinating mechanisms and take time, consensus, and intellectual contribution. The guidelines can be helpful in terms of providing a picture of how the health systems should ideally function to support the comprehensive package of interventions. However, the guidelines do not necessarily offer specific advice about how to improve the health system and “make the link” between the client and the full range of services recommended; these types of considerations have to be tackled at the national planning level.

These international guidelines typically are used to inform national ones, and national policies are also developed though multiagency coordinating mechanisms, such as national technical working groups. For example, Ethiopia has guidelines for prevention of mother-to-child of HIV that includes the minimum service delivery package for antenatal care and HIV related services, including services at the various levels and recommendations for health system inputs to support delivery [39]. Kenya appears to be the only country to have a national strategy on reproductive health and HIV integration [40].

At the subnational level, policies are created at the district and other subnational levels to guide implementation. At service delivery points, standard operating procedures define in detail procedures for implementation.

But, for most priority health outcomes and impacts, there is no guidance about what the minimum package of integrated services should be. A lack of universally agreed minimum set of interventions has been pointed to as one of the main reasons for the failure to deliver maternal, newborn and child health interventions at scale [18]. Programs for orphans and vulnerable children comprise another area where there are various recommended services for orphans and vulnerable children, but there is a lack of consensus on the minimum package, mechanisms for implementation, and empirical evidence to guide implementation [41]. There is a role for the international community to test service delivery packages then develop guidance that can be adapted by countries depending on country level priorities and epidemiology.

#### Create a theory-driven logic model

Once the vision for what priority health needs will be addressed (end in mind) through integration and how this vision will be addressed (package of services delivered at specific points of contact), the next step in the process is to create a logic model (also known as a program impact pathway). A logic model clearly defines the mechanisms for integration and implementation and visually links inputs and processes with expected health outputs, outcomes, and impacts. Inputs and process can include changes to the health system, such as changes to governance, financing, infrastructure, information and communication technologies, health workforce, and supply chain [17]. Outputs include measures of service readiness, access, and quality of care. Outcomes are measures of service coverage and risk behaviors. Impacts are improved health, equity, and efficacy.

The logic model is an important planning and operationalization document for any health program, as well as a tool for M&E [21]. Logic models are usually defined through consensus meetings that require time and intellectual contribution. The logic model serves to:

•map out in detail how the implementation of the intervention (process) is anticipated to affect health outputs, outcomes, and impacts;

•determine the policy and planning level inputs needed at all levels of the health system to carry out the implementation of the intervention;

•specify how the intervention will be carried out at all levels (community, primary, and tertiary);

•map existing donor resources and national health budgets to inputs and identify gaps;

•identify indicators to be measured every point along the chain;

•map data needs to indicators and identify gaps in health information; and

•map out partners’ roles in implementation and assign of roles and responsibilities.

While logic models are critically important tools in the evaluation of any health program, they are especially crucial in the evaluation of integrated health interventions because of the complexity of insuring that the key health system building blocks are appropriately leveraged in order to achieve maximum health impacts with maximum efficiency. These details are best defined at the national level through a dedicated process that should yield specific recommendations and assign roles and responsibilities to ensure that the inputs and funding are available to carry out planned activities and interventions.

Lack of program impact is often attributed to either a failure to fully implement the intervention or a failure by intended recipients to fully utilize all elements of the intervention. Therefore, it is important to ensure that the logic model states explicitly the program theory; in other words, that it spells out the underlying assumptions about what changes the intervention is intended affect [42]. Careful analysis of what is happening along the continuum from intervention inputs to intervention impact is involved. This analysis requires formative needs assessments, process and operational studies, and outcome and impact information.

Integrated interventions are often hard pressed to demonstrate the “value added” or to prove that integration at some process level was critical to any improvements in health outcomes or impacts. Integrated interventions involve changes in a set of activities and therefore have longer causal pathways and involve more factors that can influence the causal chain [43]. The logic model process, however, can help insure that all stakeholders share a common understanding of how the integrated intervention is to be implemented, is implemented, and how that might be expected to influence any change in downstream health indicators. Moreover, this process will help to clear any lingering questions about defining what is meant by “integration” for any given intervention that is implemented and evaluated, and thereby strengthen the evaluation and the resulting evidence base.

#### Improve the health information system

The implementation of integration, like all other types of health programming, is dependent on the functionality and skilled interlinking of all health system elements. Successfully monitoring and evaluating integrated interventions, however, is going to require particularly strong health information systems. Health information systems are the sources of data for indicators to measure inputs, processes, outputs, and outcomes and impacts defined from logic models. That information comes from censuses, birth and death monitoring, disease and behavioral surveillance, surveys, service data, mapping services, financial sources, modeling, and health research [25].

Two health information systems innovations are particularly important for integrated interventions. First, client information that is accessible to health providers and follows clients through the health system will be important for integrated interventions that rely on screening and referrals. Systems have to account for the fact that integration may include interaction between health facility and community-based services. Health information systems typically are weak when it comes to capturing information from community-based services or services provided outside the public sector. Typically, client information falls far short of this goal of good integration, but innovations such as the three interlinked patient monitoring system, family card, or electronic medical records have made large gains in this area [44-46]. Changes have already begun. In Zambia, the National AIDS Strategic Framework points to integrated reporting for prevention of mother-to-child of HIV, care and treatment, and TB as a priority [32].

Second, there is growing recognition of the need to integrate (or make “interoperable”) single sector information systems [9]. Interoperable routine health information systems are necessary for programs to be able to share information and for governments to have a complete picture of the coverage of interventions being implemented (singly or as part of an integrated service package), regardless of the sector or reporting stream from which that intervention originated [47]. Solutions to the interoperability of health information systems are twofold. First, information technology solutions serve as the platforms to combine data. Second, leadership, cooperation, and partnership guide the process, develop harmonized tools and training, and help allocate resources effectively [48]. Interoperable information systems will help monitor integrated interventions by capturing numbers of people tested, screened, treated, etc. across service delivery points (e.g., all services offering HIV testing). This is no easy task, however. Existing routine systems are often criticized for poor data quality and completeness, for their emphasis on reporting indictors at the expense of other M&E functions, and for limited data use for program management [9].

When developing monitoring and evaluation plans for integrated programs, new indicators will be needed to get some outcome indicators, such as proportions of clients receiving integrated services, met needs, service quality, and referrals and counter referrals. Caution is urged with new indicators as there is already a proliferation of indicators posing huge burdens on front line providers and yielding low quality and incomplete information [9]. Efforts will need to continue to streamline reporting mechanisms, harmonize reporting requirements, and improve the demand for information to make room for new indicators needed to monitor and evaluate integrated interventions. Clearly defined logic models will help prioritize key indicators. Further, care should be taken not to “reinvent the wheel” since there are some existing indicators to draw from (see for example [49,50]).

#### Use data in decision making

From the outset, stakeholders need to make a specific link between data collection and national strategic plans, operational plans, and program plans. Making that link requires explicit attention to determinations about what data are needed for program decision making; in other words who are the different stakeholders (e.g., ministers, district level managers, program managers, provider, etc.) and what information do they need to have in order to make timely and informed decisions which will improve programs? [48]. These questions are especially critical for the M&E of integrated service delivery programming due to the relative scantiness of the existing evidence base for decision-making. Data collected as part of a quality M&E effort around integrated programs will allow the data collection to adequately inform program decision making; to provide information for the refinement of the logic model’s inputs, processes, and indicators for integration; and to help build up the evidence base for integration efforts.

Formative assessments and situation analyses are methods that are used typically in the early implementation phases of programs to identify gaps, service needs, and barriers to intervention utilization. Process evaluation and operational study data help understand how well implementation is going and to correct any problems with implementation that could lead to a lack of effective integration of all services identified in the integrated service package. Process evaluation helps identify whether the inputs and activities defined in the logic model are being implemented as planned, in a timely manner, on a sufficient scale, and according to standards for quality [51]. Process data complement impact evaluations by informing whether the observed impact was due to the intervention or the way that the intervention was implemented [52]. Impact and outcome evaluation provide evidence for decision making, course correction, refinement, and scale up. This approach requires that intervention program managers monitor inputs, processes, outputs, and outcomes against the logic model.

Studies of integrated interventions have found positive effects on increasing access, changing behaviors, service quality, and some health outcomes, without negative cost implications [7,53,54]. Drawing general conclusions from the evidence is limited, however, as studies are modeling or pilot studies and studies were conducted in diverse settings and vary in the integrated strategy tested. Despite the increasing attention, the international community still lacks information about the effectiveness of integrated programs compared to vertical ones, the comparative efficiency of different integrated models (e.g., provider level vs. facility level vs. referrals), the effect of integration in to existing health platforms, such as HIV, and the effectiveness of programs at scale up across contexts [7,55,56].

Integrated interventions pose a number of challenges to evaluation study designs. While randomized control trials are the preferred design for impact evaluation, they require relatively large sample sizes, adequate control groups, and exposure to a well-defined primarily biomedical intervention [43,57]. Integrated interventions will decrease the ability to randomize because they may act at the national level, increase the difficulty of defining and measuring exposure to the intervention, increase the complexity of the causal pathway and the number of mediating factors, affect a wider range of outcomes, and vary in intensity of implementation [12]. Hypothesized causal pathways of influence (strong logic models/program implementation pathways) and strong process and costing data (acquired through strong, well-functioning health information systems and process evaluations) can help overcome some of these limitations and identify where the gaps are in implementation, explain observed impact results, inform how to scale up interventions, and estimate how much the successful implementation of integrated programs will cost [21].

## Summary

Integrated interventions are fundamentally client centered and seek to improve the effectiveness and efficiency of providing a continuum of care to improve the health and well-being of those clients. Integration is a core Global Health Initiative principle and is viewed as a means to achieving critical public health goals, including the Millennium Development Goals [58]. In this paper, we have attempted to apply basic M&E questions for national level M&E systems and M&E best practices embodied in existing frameworks to define a systematic approach at the country level for prioritizing integrated interventions, developing an M&E plan, and establishing an evidence base to inform those who manage these investments. The approach we outline is borne out of and consistent with single-sector M&E frameworks that employ a stepwise approach to M&E to ensure that information is available to inform decisions throughout the cycle of planning, monitoring, data collection, analysis, revision, and evaluation. The steps identified have been discussed in the light of the special considerations and modifications that make them necessary and effective for the M&E of integrated health programming.

The approach is grounded by first defining the health impacts integration is intended to affect. The mutual goal of designing, implementing, and scaling up interventions to improve a particular health outcome or impact is the “glue” that holds together disparate interests, services, and sectors. Second, it calls for a thorough understanding of the key point of contacts for adding new activities and services to maximize public health impact. A third advantage is the role for all levels of policy in creating “essential packages” of integration for all major client presentations. International minimum guidelines draw on the global evidence base and expert opinion. This international guidance informs individual country strategies and guidelines, including appropriate modifications to policies and standard operating procedures guiding practice at the service delivery level. Fourth, this paper describes the role of logic models to outline the plausible causal pathways and define the inputs, roles and responsibilities, indicators, and data sources across the health system. Finally, we recommend improvements to the health information system and in data use to ensure that data are available to inform decisions.

There are some notable differences and similarities between this approach and approaches to single sector (i.e., vertical, disease oriented) M&E. In both cases the country’s epidemiology informs the strategic planning and M&E goals and targets. The difference compared with single sector M&E is that this approach requires a more systematic assessment of the service delivery system entry points; the set of activities that need to be added; and the national and operational guidelines that need to be tested, revised, or created. Most importantly single sector M&E approaches are often driven by institutions external to the national governments, such as by donors, and those external institutional priorities might not reflect those of each country [48]. This paper’s approach requires that program planning and M&E systems be determined based on national level priorities. While this may increase the likelihood that the programmatic response is tailored, it does not mean that donor funding will correspond with the country’s priorities. Further, there may be human and financial resource constraints that limit application of the approach.

Because the perspective of this approach is at the country level, it is consistent with calls for greater country ownership [1,4]. It is the country and its institutions that coordinate between global and national partners and where international partners bring their agendas in line with national ones, instead of vice versa [59]. However, it will be a challenge to overcome the vertical, single sector approach to program planning and M&E and adopt practices that respond to country priorities and reflect a more integrated approach.

The approach represents the ideal, and thus it faces certain challenges. Despite a commitment in the public health community to the kind of logical and stepwise approach to M&E that we have recommended in this paper, execution of this kind of approach often falls short even for single service interventions, particularly at the country level where logic models and program impact pathways may be incomplete. Applying the framework requires making serious progress in overcoming parallel health information systems. Strengthening health information systems in general and ensuring that routine data systems are “interoperable” will result in high costs to donors, governments, and partners; the value of which is hard to communicate to donors and politicians [59]. Successful application of this framework also requires collaboration and cooperation amongst stakeholders of all types and at all levels of public health policy, programming, and practice from the international, national, sub-national, and service delivery level. This framework must be taken to heart by the entire public health community and not just by single actors if it is to be successful in reframing thinking about how integration is implemented, monitored, and evaluated. Evaluation of integrated interventions will also require increased tolerance for the inability to attribute changes to a particular program or donor and for negative or unanticipated outcomes.

More experience is needed to understand better how the M&E approach we outline will be useful in the realities of countries. While there is a role for rigorous studies of integrated models, we argue that more emphasis is needed on documenting the process of designing and implementing integrated models at the national level that take into account the national epidemiological, health systems, social and political factors. The question shifts from “what is the effectiveness of the particular integrated model (compared with non-integrated)?” to “what is the effectiveness of integrated model designed for a specific context?” At the same time, the process by which the model was designed and implemented is thoroughly documented, in order to inform adaptation of the process (rather than a particular model) in another context. Continued rigorous testing of integrated models across various contexts will eventually, through systematic reviews, yield conclusions about the characteristics of the interventions and contexts that are favorable for implementation. But the complexity of integration and sheer number of possible service delivery combinations, health system improvements, and implementation contexts requires a different approach. Public health professionals will continue to build and fill gaps in the evidence base for what is new and what works in integration to promote the health and well-being of global communities, and the M&E approach that we have outlined can help contribute to that ongoing effort.

There are several development trends emerging which increase the likelihood of systematic planning and implementation of integrated intervention strategies, and the potential success of an M&E approach to integration such as the one we propose here. The aforementioned unprecedented and widespread international promotion of integration and country ownership as core principles to improve health outcomes is one of these emerging trends. Another is the attention being paid to health system strengthening in general, and not just for the purposes of integration. Strong health systems are required if moving to a more complex integrated, rather than vertical, approach to health care is to be successfully undertaken. There are also calls for more unified M&E systems, again in general and beyond the needs of specific integration efforts, to allow evaluation efforts benefit from and capitalize on all health information emerging from monitoring and reporting systems [24,60].

## Endnotes

^a^http://www.internationalhealthpartnership.net/en/

## Abbreviations

M&E: Monitoring and evaluation; PEPFAR: U.S. President’s Emergency Plan for AIDS Relief; TB: Tuberculosis; UNAIDS: Joint United Nations Programme on HIV and AIDS; USAID: United States Agency for International Development; WHO: World Health Organization.

## Competing interests

The authors declare the have no competing interests.

## Authors’ contributions

HR and ES made contributed to the manuscript conception and design, drafted and revised the manuscript, and read and approved the final manuscript.

## Authors’ information

HR is Deputy Director of HIV and AIDS and Other Infectious Diseases and ES is senior technical specialist-HIV and AIDS for the MEASURE Evaluation project, a 5-year, USAID funded project supporting the monitoring and evaluation of population, health and nutrition worldwide, based in the Carolina Population Center at The University of North Carolina at Chapel Hill.

## Pre-publication history

The pre-publication history for this paper can be accessed here:

http://www.biomedcentral.com/1472-6963/13/168/prepub
